# Long-term neurological outcome of a cohort of 80 patients with classical organic acidurias

**DOI:** 10.1186/1750-1172-8-148

**Published:** 2013-09-23

**Authors:** Mathilde Nizon, Chris Ottolenghi, Vassili Valayannopoulos, Jean-Baptiste Arnoux, Valérie Barbier, Florence Habarou, Isabelle Desguerre, Nathalie Boddaert, Jean-Paul Bonnefont, Cécile Acquaviva, Jean-François Benoist, Daniel Rabier, Guy Touati, Pascale de Lonlay

**Affiliations:** 1 Centre de Référence des Maladies Héréditaires du Métabolisme, Hôpital Necker-Enfants Malades, APHP, Université Paris Descartes, Institut Imagine, Paris, France; 2 Service de Biochimie métabolique, Hôpital Necker-Enfants Malades, U747, Inserm - Université Paris Descartes, Paris, France; 3 Service de Neurologie, Hôpital Necker-Enfants Malades, APHP, Université Paris Descartes, Paris, France; 4 Service de Radiologie Pédiatrique, Hôpital Necker-Enfants Malades, APHP, Université Paris Descartes, Institut Imagine, Paris, France; 5 Service de Génétique Médicale, Hôpital Necker-Enfants Malades, APHP, Université Paris Descartes, Institut Imagine, Paris, France; 6 Service de Maladies Héréditaires du Métabolisme, Centre de Biologie et Pathologie Est, Bron, France; 7 Centre de Référence des Maladies Héréditaires du Métabolisme, Service de Biochimie, Hôpital Robert-Debré, APHP, Paris, France

**Keywords:** Organic aciduria, Propionic aciduria, Methylmalonic aciduria, Isovaleric aciduria, Neurological evolution, Long-term prognosis, Mitochondrial dysfunction

## Abstract

**Background:**

Classical organic acidurias including methylmalonic aciduria (MMA), propionic aciduria (PA) and isovaleric aciduria (IVA) are severe inborn errors of the catabolism of branched-chain amino acids and odd-numbered chain fatty acids, presenting with severe complications.

**Methods:**

This study investigated the long-term outcome of 80 patients with classical organic aciduria (38 with MMA, 24 with PA and 18 with IVA) by integrating clinical, radiological, biochemical and genetic data.

**Results:**

Patients were followed-up for a mean of 14 years [age 3.3-46.3 years]. PA included a greater number of patients with abnormal neurological examination (37% in PA, 24% in MMA and 0% in IVA), lower psychometric scores (abnormal evaluation at age 3 years in 61% of patients with PA versus 26% in MMA and 18% in IVA) and more frequent basal ganglia lesions (56% of patients versus 36% in MMA and 17% in IVA). All patients with IVA presented a normal neurological examination and only 1/3 presented cognitive troubles. Prognosis for MMA was intermediate. Biochemical metabolite analysis excluding acute decompensations revealed significant progressive increases of glycine, alanine and glutamine particularly in PA and possibly in MMA but no correlation with neurological outcome. A significant increase of plasma methylmalonic acid was found in MMA patients with intellectual deficiency (mean level of 199 μmol/L versus 70 μmol/L, p < 0.05), with an estimated significant probability of severe outcome for average levels between birth and age 6 years above 167 μmol/L. Urinary 3-hydroxypropionate (3-HP) levels were significantly higher in PA patients with intellectual deficiency (mean level of 68.9 μmol/mmol of creatinine versus 34.6 μmol/mmol of creatinine, p < 0.01), with an estimated significant probability of severe outcome for average levels between birth and age 6 years above 55 μmol/mmol. As for molecular analysis, prognosis of MMA patients with mutations involving the *MMAA* gene was better compared to patients with mutations involving the *MUT* gene.

**Conclusion:**

Propionic aciduria had the most severe neurological prognosis. Our radiological and biochemical data are consistent with a mitochondrial toxicity mechanism. Follow-up plasma MMA and urinary 3-HP levels may have prognostic significance calling for greater efforts to optimize long-term management in these patients.

## Background

The classical organic acidurias are branched-chain amino acids disorders involving intermediate metabolism. Depending on the enzymatic block, three major organic acidurias have long been distinguished: isovaleric aciduria (IVA) designates an isovaleryl-coA deshydrogenase defect on the leucine degradation pathway secondary to mutation in the *IVD* gene, propionic aciduria (PA) results from a defect in propionyl-coA carboxylase encoded by the *PCCA* or *PCCB* genes, and methylmalonic aciduria (MMA) is caused by mutations in the *MUT* gene, encoding the methylmalonyl-CoA mutase (MCM), or more rarely in genes encoding the coenzyme adenosylcobalamin of MCM. MCM converts methylmalonyl-CoA into succinyl-CoA, an intermediate of the tricarboxylic acid cycle, the major source of NADH used by the mitochondrial respiratory chain. Two of these diseases, PA and MMA, affect the pathway common to valine, isoleucine, methionine and threonine metabolism.

Organic acidurias (OA) usually present as acute metabolic distress at birth with coma associated with dehydration when the enzymatic deficiency is complete, or later in life with recurrent ketoacidotic episodes, psychomotor retardation and chronic vomiting when the deficiency is partial [1,2]. As propionate is produced by the catabolism of branched-chain amino acids, fatty acids with a carbon odd-chain and the intestinal flora, the treatment is based on a strict low-protein diet associated with sufficient caloric intake, carnitine and antibiotics. However, despite therapeutic improvements over the last 20 years, overall outcome of patients with OA remains unsatisfying. Reports are increasing of long-term complications, such as neurological disorders by degeneration of the basal ganglia, progressive renal failure in MMA, cardiomyopathy in PA and acute pancreatitis in all [3-5].

Specific changes in the levels of urinary and plasma metabolites are the hallmark of the classical forms of the diseases including ketoacidosis, hyperlactatemia, hyperamoniemia, cytopenia in variable proportions. In urine, several organic acids are quite specific for diagnosis, particularly 3-hydroxypropionate and methylcitrate in PA, methylmalonate in MMA and isovalerylglycine in IVA. Enzymatic and genetic analyses confirm the diagnosis.

The pathophysiology of these disorders is not clearly understood and the proposed mechanisms are complex. Metabolite accumulation upstream of the enzymatic block triggers a systemic endogenous intoxication. Furthermore, the metabolic pathway involved in classical organic acidurias contributes to acetyl-coA and succinyl-coA formation, which are required for the tricarboxylic acid cycle. Thus, there is an energetic deficit by mitochondrial dysfunction secondary to substrate insufficiency and specific toxic metabolite accumulation including 3-hydroxypropionate [1].

The long-term neurological prognosis of these disorders depends on the severity of the disease, the delay of diagnosis and probably specific biochemical and genetic parameters. In particular, several studies attempting the delay to describe the neurological evolution of organic acidurias were hampered by the wide phenotypic variability even in a homogenous genetic population [6]. In order to better delineate the long-term neurological outcome of patients with organic acidurias, we analyzed clinical, radiological, biochemical and genetic parameters of a large cohort of patients.

## Methods

### Patient cohort

Inclusion criteria was a positive biochemical diagnosis of propionic, methylmalonic or isovaleric aciduria in a cohort of patients followed up at Necker-Enfants Malades hospital from 1991 to 2012 and managed with similar chemical and diet therapy. Exclusion criteria were an age less than 3 years old at the time of study, death before age 3 years or insufficient clinical data.

Neonatal onset refers to patients diagnosed within the first month. “Late onset” patients were all those diagnosed thereafter.

### Neurological evaluation

Neurological evaluation was based on retrospective clinical data available in medical reports and was completed by radiological data (brain scanner and magnetic resonance imaging).

### Psychometric evaluation

Each patient’s psychometric evaluation was supported by school data and psychometric tests depending on the age of the patient and the evaluation year (Brunet Lezine from 0 to 2 ½ years old, Brunet Lezine with complementary test from 2 to 6 years old, WPPSI-III from 3 to 7 years old, NEMI from 4 to 12 years old and WISC-R and WISC-IV from 6 to 17 years old). Heterogeneity of the tests made it impossible to carry out a precise comparative study, thus we distinguished four distinct clinical groups based on developmental or intellectual quotient and autonomy level: group 0 with IQ > 85 or DQ > 90 in normal school, group 1 with IQ = 71-85 or DQ = 76-90 in normal school with specialized assistance for children in difficulties, group 2 with IQ = 51-70 or DQ = 56-75 in specialized classroom within normal school presenting mild intellectual deficiency and group 3 with IQ ≤ 50 or DQ ≤ 55 in specialized establishment presenting moderate to severe intellectual deficiency.

Neurological evaluation was performed at 1, 3, 6, 11 and 18 years old leading to the definition of four groups in term of cognitive evolution: group A with autonomy and good evolution (group 0 or 1 maintained from age 1 to 18 years), group B with improvement of psychometric evaluation over time (transition from initial classification into group 2–3 to classification into group 0–1), group C with worsening of psychometric evaluation (from group 0–1 to group 2–3) and group D with poor outcome from the outset and absence of autonomy.

### Acute decompensations

Five different clinical conditions of variable severity were defined and reported on different periods from 0 to 3, from 3 to 6, from 6 to 11 and from 11 to 18 years old: 1) conditions at risk of acute decompensation needing an emergency diet at home; 2) conditions at risk of acute decompensation needing hospitalization for emergency diet by naso-gastric tube or intravenously without biological findings; 3) clinical and biological decompensation including pH < 7.30, bicarbonatemia < 18 mmol/L, ammoniemia > 70 μmol/L or neurological trouble, 4) severe acute decompensation with intensive care and 5) severe acute decompensation with haemofiltration.

### Biochemical analysis

Standard biochemical blood analysis included ammoniemia, lactate, amino acids (with particular emphasis on glycine, alanine, glutamine) and propionylcarnitine during basal state and acute decompensation, as well as pH, bicarbonatemia during acute decompensation. Specific metabolites included urinary 3-hydroxypropionate, propionylglycine, tiglyglycine and methylcitrate for PA, plasma and urinary methylmalonate for MMA and urinary isovalerylglycine for IVA. Amino acids were assayed by ninhydrin colorimetry (Jeol Aminotac Analyzers) and organic acids by gas chromatography–mass spectrometry (Varian Saturn-2000).

### Genetic analysis

Organic acidurias are recessive autosomal genetic diseases. Mutations in the *PCCA* (Propionyl CoA Carboxylase, alpha subunit, GenBank:NG_008768) and *PCCB* (Propionyl CoA Carboxylase, beta subunit, GenBank:NG_008939) genes for *PA*, *MUT* (Methylmalonyl CaA mutase, GenBank:NG_007100), *MMAA* (Methylmalonic Acidemia, cblA type Gene, GenBank:NG_007536) and *MMAB* (Methylmalonic Acidemia, cblB type Gene, GenBank:NG_007096) genes for MMA and *IVD* (Isovaleryl Coa dehydrogenase, GenBank:NG_011986) for IVA were identified after PCR amplification of patient’s DNA and dideoxynucleotide sequencing using the BigDye terminator cycle sequencing kit (Applied Biosystems, Foster City, CA, USA) and analysis with ABI Prism 3100 DNA sequencer.

### Statistical analysis

The statistical analysis consisted in comparison of means by the two-tailed Student test with significance accepted for p < 0.05. Comparisons involving more than two groups were performed with one-way ANOVA followed by the Tukey's post-hoc test or two-way ANOVA. Logistic regression was performed with the *bayesglm* module in R.

## Results

### Demographic analysis of the cohort of patients

**Figure 1:**
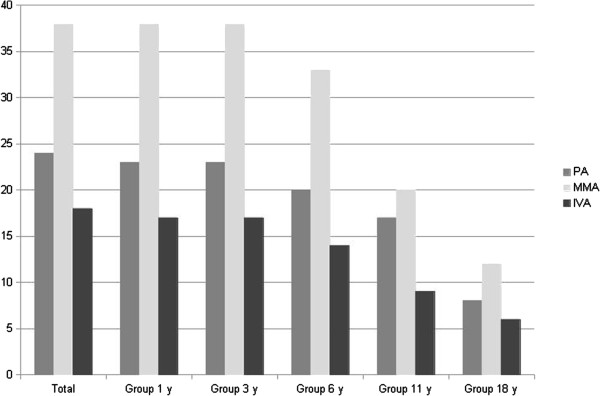
**Number of patients with available clinical data according to age.** PA: propionic aciduria, MMA: methylmalonic aciduria, IVA: isovaleric aciduria; y: years.

The cohort included 80 patients from 74 families, 46 boys and 34 girls, born after 1990 and being alive after age 3 years, including 24 PA, 38 MMA and 18 IVA. Clinical follow-up of the cohort is shown in Figure 1. Mean age was 14 years and the median was 12.3 years [3.3-46.3 years old]. 58% of patients have been diagnosed during the first month (median current age, 11.4 years old). 42% of patients had a presentation from 1 month to 18 years old (median current age, 13.2 years old). Metabolic dysfunction led to liver transplantation for one PA patient and kidney transplantation for two MMA patients. Concerning MMA patients, only two sibling patients were sensitive to B12 supplementation. Thus, no correlation could be established with this series. Finally, one patient with PA died at 19 years old of an accidental fall, one with MMA died at 17 years old of a gastrointestinal bleeding after hernia surgery, and two patients with MMA died following an acute metabolic episode at 11 and 7 years old respectively.

### Clinical neurological and psychometric evaluation

**Figure 2:**
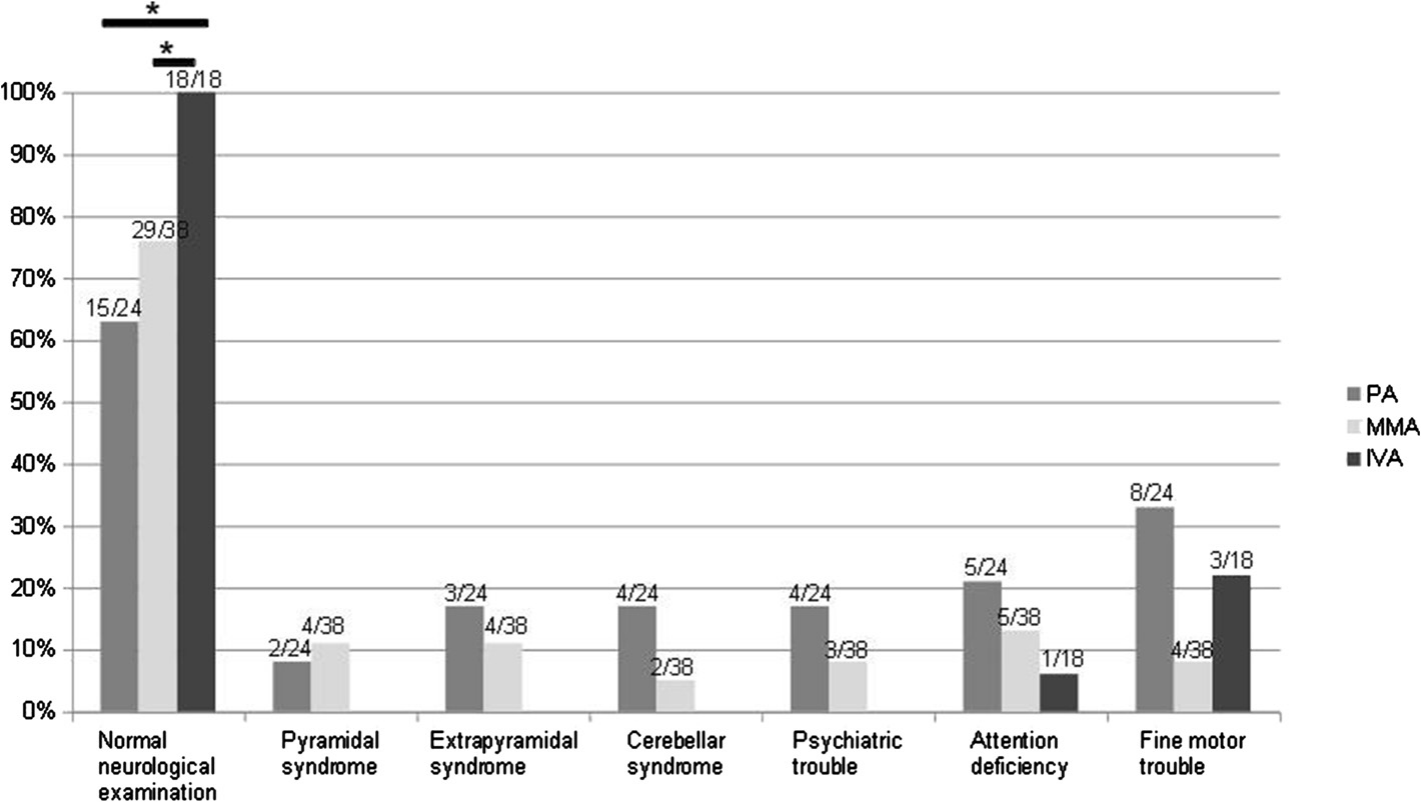
**Neurological examination data for patients with propionic aciduria (PA), methylmalonic aciduria (MMA) and isovaleric aciduria (IVA) at the last examination.** Median age of PA patients, 13 years; median age of MMA patients, 10.9 years; median age of IVA patients, 11.9 years.

Clinical neurological examination was considered normal in 63% of PA (median age of 13 years old [3.8-46.3 years old]), 76% of MMA (median age of 10.9 years old [3.3-33.4 years old]) and 100% of IVA (median age of 11.9 years old [3.4-25.3 years old]) (p < 0.01 between IVA and MMA or PA, see Figure 2). Among the 18 patients with abnormal neurological examination, 33% had a pyramidal syndrome, 44% had an extrapyramidal syndrome, 33% a cerebellar syndrome and 28% of the patients required assistance to walk (44% for PA, 11% for MMA and none for IVA).

Moreover, seven patients were found with psychiatric disorders. Among PA, two teenagers presented with a psychotic disorder with hallucinations and disorganized behavior that was triggered by a moderate metabolic decompensation and was controlled by antipsychotic treatment within a few months. Such therapy may need to be adapted in case of cardiomyopathy or long QT syndrome. A third PA patient had a depressive and sleep disorder and one had an anxiety disorder. Among MMA, two patients had behavioral disorder and one patient had a personality disorder with anxiety. Most of these patients were treated by psychotherapy and by conventional drugs. Among PA, one patient followed up to 46 years old had memory trouble with ictus amnesic episodes. 6 to 21% of patients had attention trouble and 8 to 33% of patients had fine motor trouble disturbing handwriting and requiring rehabilitation.

**Figure 3:**
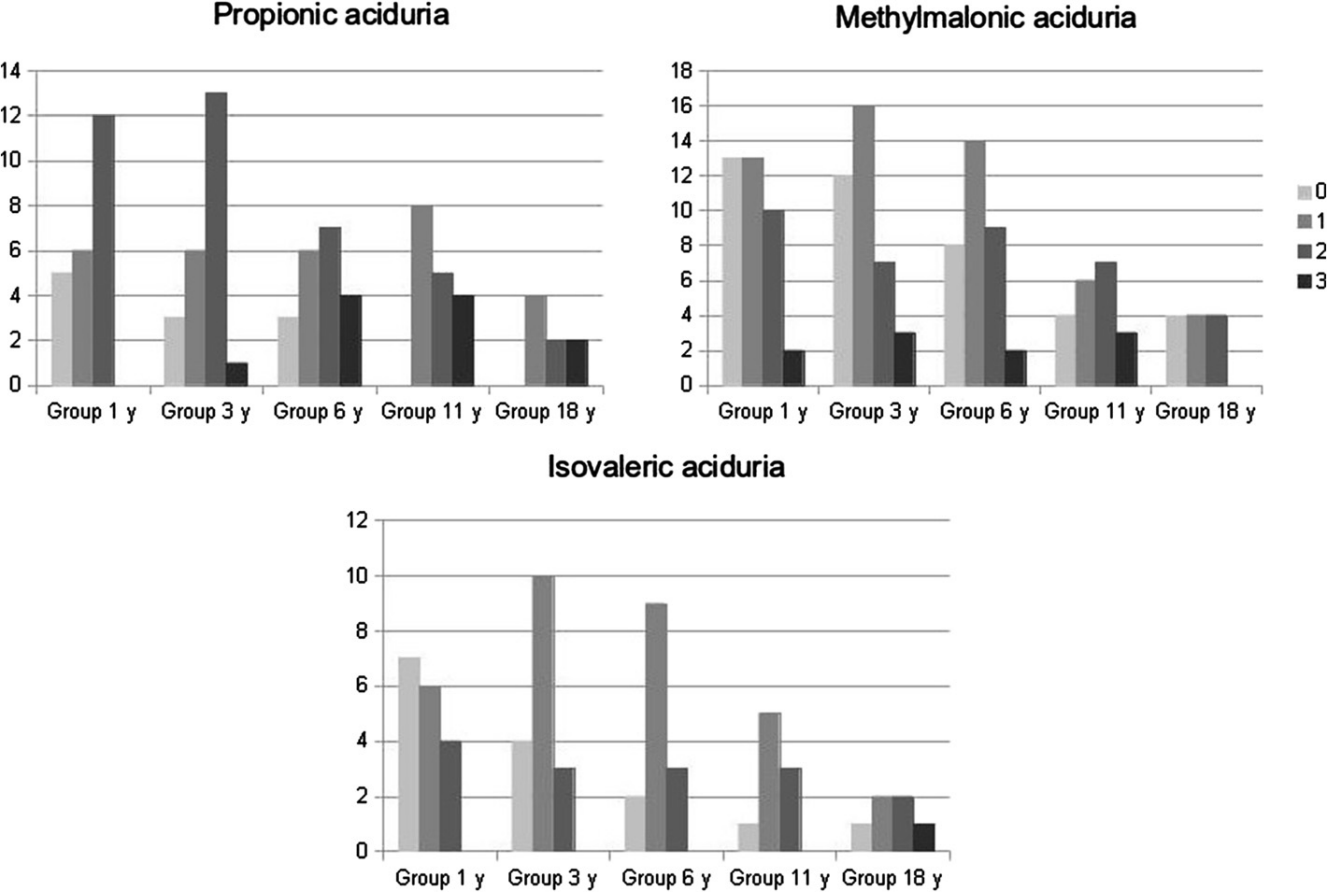
PA, MMA and IVA patients repartition according to psychometric tests at different ages (normal 0–1, intellectual deficiency 2–3).

**Figure 4:**
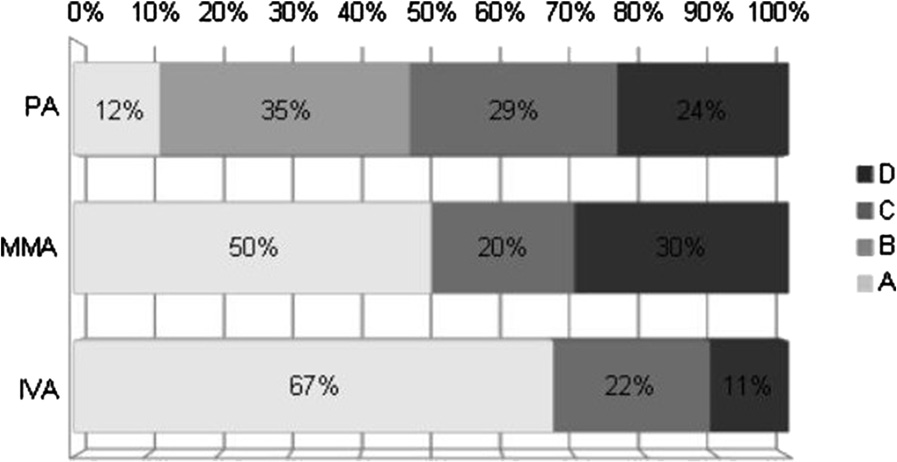
Cognitive evolution for patients diagnosed with organic aciduria from 3 to 11 years old (A normal, B improvement, C worsening, D intellectual deficiency from the onset).

Psychometric evaluation at different times highlighted a specific pattern attendant on the type of organic aciduria (Figures 3 and 4). The score was considered as normal (groups 0 and 1) in 39% and 47% of patients with PA at ages 3 and 11 years respectively, in 74% and 50% of patients with MMA at ages 3 and 11 years respectively, and 82% and 67% of patients with IVA at ages 3 and 11 years respectively. Interestingly, at age 11 years, group 0 included only a few patients: 4/20 for MMA, 1/9 for IVA and 0/17 for PA. By contrast, at this age, 29% of PA (12/17), 25% of MMA (15/20) and 11% of IVA (1/9) had deficiency in expression and comprehension skills.

PA patients were neurologically more severely affected than MMA and IVA at 3 years of age, with 61% of PA patients in group 2 or 3 compared to 26% for MMA and 18% for IVA (p < 0.01 between PA and MMA or IVA, ANOVA test). The outcome was good for only 12% of patients with PA comparing to 50% MMA patients and 67% IVA patients (group A). Of note, the data indicated psychometric improvement in 35% of PA patients from group B and not in other organic aciduria. Then, at 11 years old, groups A/B and C/D proportion was about the same among the three organic acidurias with a better prognosis for IVA. Furthermore, cognitive regression concerned 20 to 29% patients for all three organic acidurias (group C at 11).

### Correlation between age of onset and neurological examination

**Figure 5:**
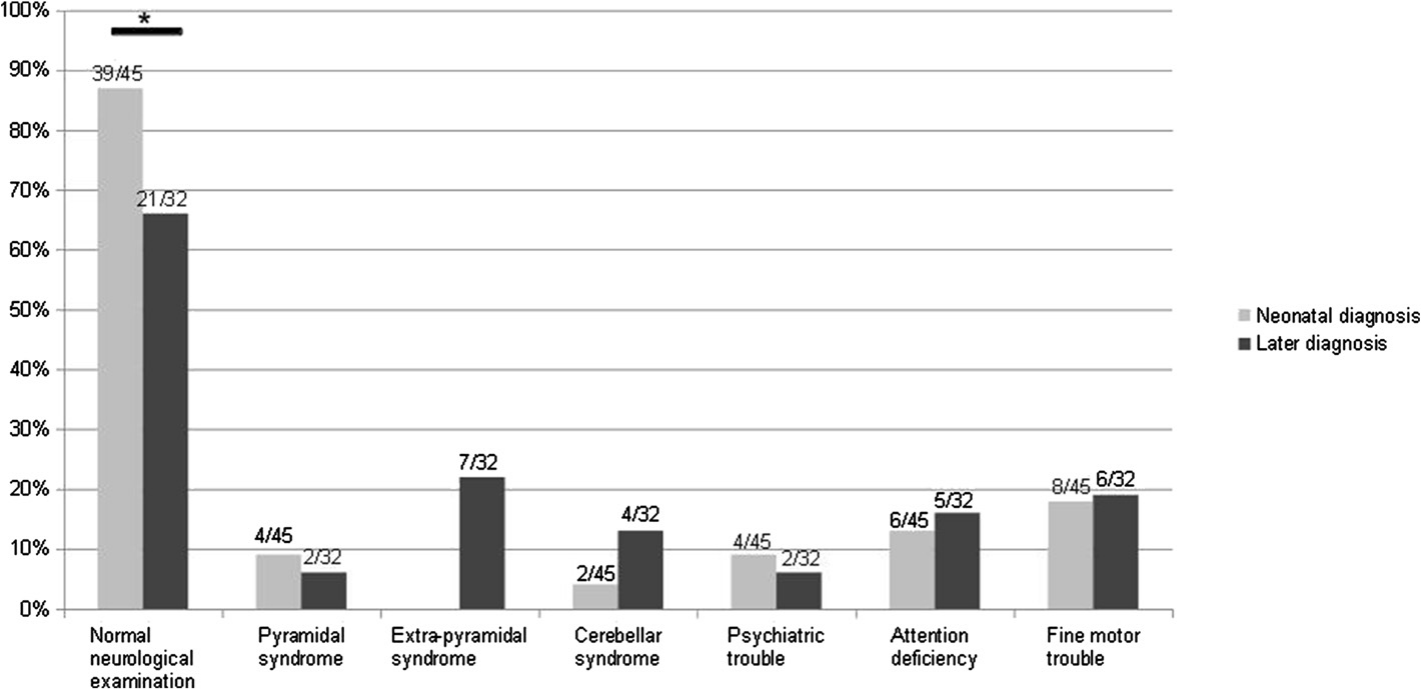
**Age at onset and neurological examination correlation.** Neonatal diagnosis (45 patients): between 0 and 30 days of life, later diagnosis (32 patients): median age 5.9 months [31 days-18 years].

Neurological examination was normal for 87% of patients with neonatal diagnosis and for 66% of patients diagnosed later (p < 0.05, see Figure 5). Both groups had pyramidal syndrome, cerebellar syndrome, psychiatric troubles, attention deficiency or fine motor troubles in insignificant different proportions. Only late onset diagnosis patients had an extrapyramidal syndrome, with frequent difficulty to walk: 3/4 PA and 1/4 MMA required assistance to walk.

### Correlation between date of onset and neurological data

Between 1991 and 2000, the neonatal resuscitation was less effective than after year 2000 and nocturnal nutrition by nasogastric tube was not systematic at that time contrary to current recommendations. Therefore, we reanalyzed data in two subgroups, depending on whether diagnosis was done before or after 2000. A fraction of 48% of the patients was diagnosed before 2000 with similar proportions among the different organic acidurias. Neurological examination was normal for 68% of patients who were diagnosed between 1991 and 2000 (50% of PA, 68% of MMA, 100% of IVA) and for 86% of patients diagnosed after year 2000 (75% of PA, 84% of MMA, 100% of IVA, p = 0.07, not significant). Psychometric tests showed no significant difference between the two groups: IQ was normal at 6 years old for 50% of patients diagnosed between 1991 and 2000 and 71% of patients diagnosed after 2000 (p = 0.08, not significant). Therefore, there was a non-significant trend toward better neurological scores (neurological examination and psychometric tests) for patients diagnosed after 2000, consistent with improved management.

### Brain imaging evaluation

**Figure 6:**
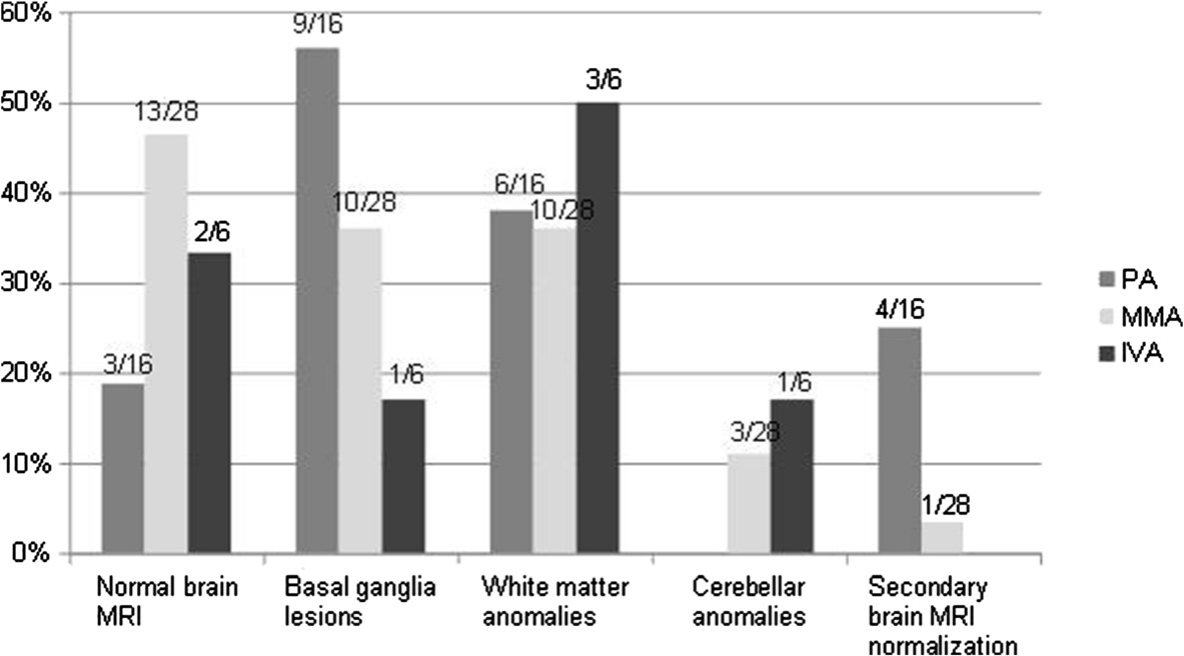
**Brain MRI data for 16 PA patients, 28 MMA patients and 6 IVA patients; median 13.1 years.** Secondary brain MRI normalization performed in 5 patients with a follow-up of 5 years.

Imaging was available for 50/80 patients including 16/24 PA, 28/38 MMA and 6/18 IVA. Brain MRI was normal for 19% of PA, 46% of MMA and 33% of IVA when available (see Figure 6). Abnormalities included basal ganglia lesions for 56% of PA, 36% of MMA and 17% of IVA, notably involving the striatum and the globus pallidum, white matter anomalies for 38 to 50% and cerebellar anomalies for three MMA and one IVA. For five patients, normalization of basal ganglia lesions and white matter anomalies were observed at a distance from the acute episode. Furthermore, two patients PA and three patients MMA had increased lactate concentration in basal ganglia. Magnetic resonance spectroscopy was not performed for the other patients.

### Brain imaging and neurological examination correlation

Among 62 patients with normal neurological examination, 30 patients had available brain imaging: the pictures were normal for 47% of patients, and showed white matter anomalies for 40%, basal ganglia anomalies for 17% and cerebellar abnormalities for 10%.

Among the six patients with pyramidal syndrome, two had normal brain MRI, three had basal ganglia anomalies, two had white matter anomalies and one cerebellar atrophy. Among the seven patients with extra-pyramidal syndrome, two had a normal brain MRI, four had basal ganglia anomalies and two had white matter anomalies. Of the four patients with cerebellar syndrome, two had a normal MRI and two had white matter anomalies. There was no correlation between long-term psychometric test evolution and brain anomalies.

### Acute decompensations

**Figure 7:**
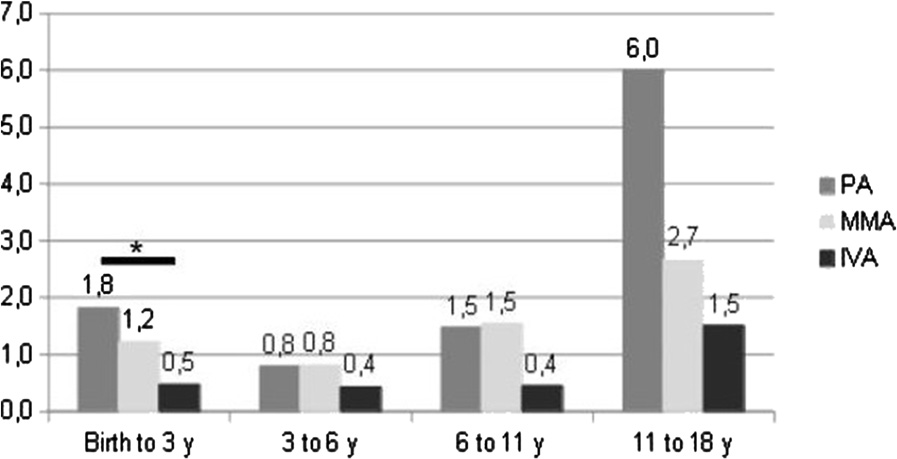
Average number of acute decompensations by patients according to age, except the initial episode.

Excluding initial decompensation, PA patients underwent an average of 1.8 acute decompensations from birth to age three years [0–8] whereas IVA patients had only 0.5 [0–2] and MMA patients 1.2 [0–7] (see Figure 7); the difference between PA and IVA was significant (p < 0.05; we did not count the initial decompensation). For the three organic acidurias, we found two frequency peaks of decompensations, from birth to three years old and from eleven to eighteen years old. Furthermore, the number of patients requiring haemofiltration for one of the acute decompensations including the initial episode was 6/24 for PA (two patients required two and three haemofiltrations respectively), 7/38 MMA and 1/8 IVA. Except for one of the seven MMA patients, haemofiltration was performed only at the first decompensation.

### Biochemical profile outside of acute decompensations

**Figure 8:**
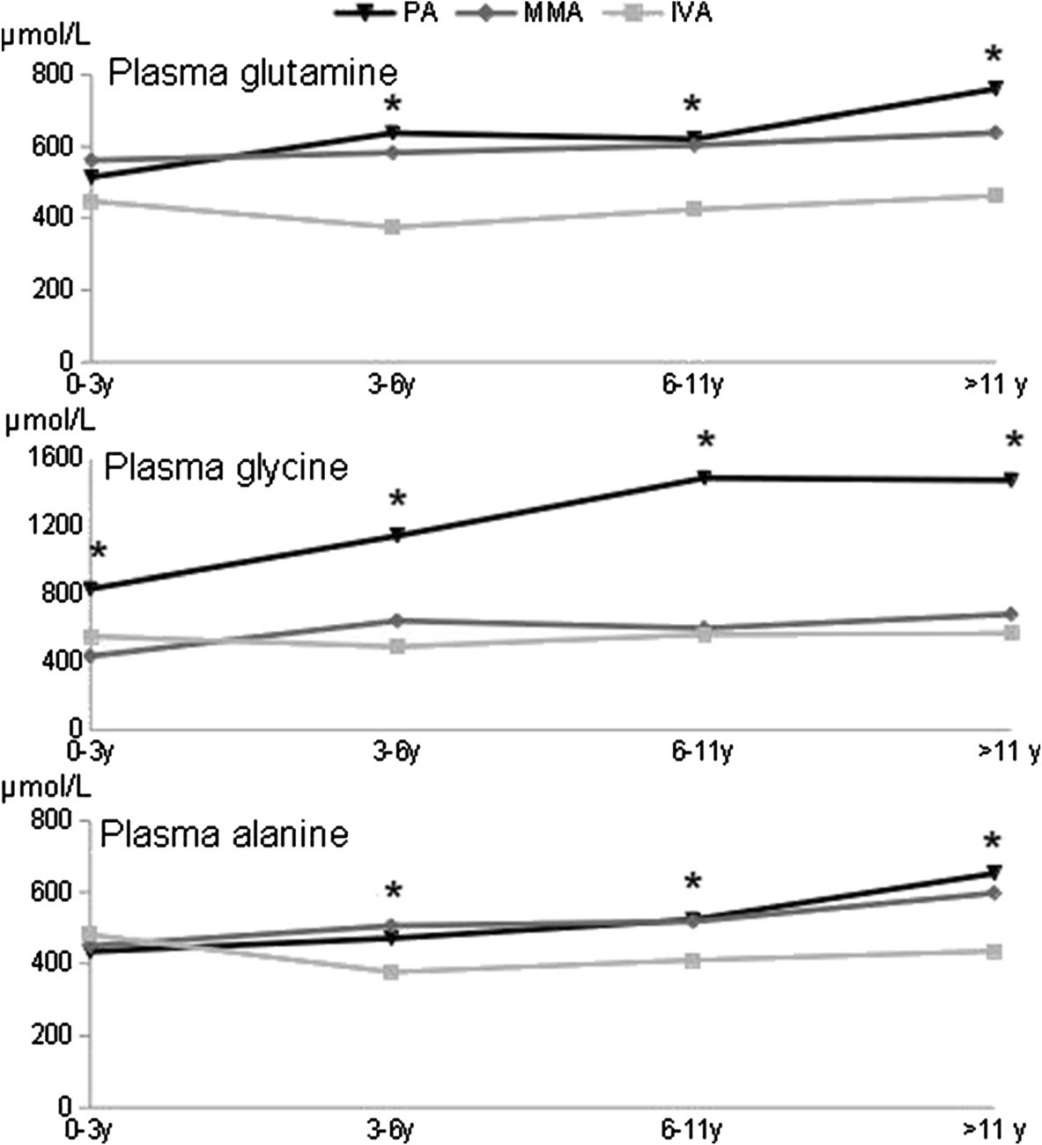
Evolution of the amino acid levels according to age by each disease (*: p-values : < 0.01).

Plasma amino acid levels differed significantly with disease status and age, showing a highly significant interaction for glycine (p = 0.0001; Table 1A). Indeed compared to MMA and IVA, the pattern of variation of glycine was strikingly specific to PA, showing a sharp increase at an early age and a high plateau at about 11 years (Additional file 1, Figure 8). In addition, the two other tested amino acids, glutamine and alanine, showed highly significant increases with age specifically in PA, whereas variations were essentially borderline in MMA, and undetectable or not progressive with age in IVA (Figure 8 and Table 1B). There was no correlation between plasma glycine, glutamine and alanine levels and the neurological, psychometric evolution and brain anomalies for any age group.

**Table 1:** Plasma amino acid levels in relation to disease and age

**1A. Joint effect of disease and age on plasma amino acid levels**			
	**Disease**	**Age**	**Age and disease interaction**
**Plasma alanine**	0.0010**	0.0069**	0.027*
**Plasma glutamine**	0.0000***	0.0012**	0.030*
**Plasma glycine**	0.0000***	0.0000***	0.0001***
**1B. Effect of age on plasma amino acid levels for each disease**			
	**IVA**	**MMA**	**PA**
**Plasma alanine**	0.051	0.068	0.0041**
**Plasma glutamine**	0.17	0.39	0.0005**
**Plasma glycine**	0.22	0.011*	0.0000***

A: Joint effect of disease status and age on plasma amino acid levels (two-way ANOVA, p-values). B: Effect of age on plasma amino acid levels for each disease (one-way ANOVA, p-values). To enhance readability, p-values are rounded following the first non-zero digit or at total four digits. Significance cutoffs for p-values: * < 0.05; ** < 0.01; *** < 0.001.

Organic acid levels average over the first six years was significantly associated with neurological prognosis for MMA and PA. Indeed, plasma methylmalonic acid was 70 μmol/L for MMA group A/B and 199 μmol/L for MMA group C/D (p < 0.05). By logistic regression, the odds ratio for severe outcome (groups C/D rather than A/B) was 4.3 (95% confidence interval: 1.1-16.3) for every two-fold multiplicative increase of average plasma MMA levels over the first 6 years, and the risk of severe outcome was significant for values greater than or equal to 167 μmol/L (probability: 0.67; 95% confidence interval: 0.51-0.84).

As for urinary 3-hydroxypropionate, the average level over the first 6 years was 34.6 μmol/mmol of creatinine for PA group A/B and 68.9 μmol/mmol of creatinine for PA group C/D (p < 0.01). Odds ratio for severe outcome was 5.1 for every two-fold multiplicative increase of urinary 3-hydroxypropionic acid levels averaged over the first 6 years. The risk of severe outcome was significant for values greater than or equal to 55 μmol/L (probability: 0.64; 95% confidence interval: 0.51-0.77)

Other metabolites were not correlated with clinical evolution, notably urinary isovalerylglycine for IVA (1556 μmol/mmol of creatinine), plasma propionylcarnitine level for MMA (45.4 μmol/L), lactatemia (2 mmol/L) and ammoniemia (54 μmol/L) for PA, MMA and IVA.

### Genetic analysis

The genotype was known for 49/80 patients (see Table 2). Two PA patients had mutation in *PCCA* (one stop, one missense and one splice mutations) and seven in *PCCB* (five stop and five missense mutations), eight MMA patients had mutation in *MMAA* (seven stop and one missense mutations), one in *MMAB* (stop mutation) and 29 in *MUT* (nine stop and sixteen missense mutations including two recurrent mutations p.Ala191Glu for 19% and p.Asn219Tyr for 13%) and five IVA patients had mutation in *IVD* (four missense mutations).

**Table 2:** List of mutations (nucleotidic or amino acid change depending on the patients) for patients diagnosed with PA, MMA and IVA and neurological evaluation

**Patient**	**Gene**	**Allele 1**	**Allele 2**	**Cognitive evolution at 11 y**	**Neurological examination anomalies**	**Brain MRI anomalies**
1	*PCCA*	c.1284 + 4A > T	p.Arg430X	A	no	
2	*PCCA*	p.Ala138Thr	p.Ala138Thr	B	yes	yes
3	*PCCB*	p.Gln147GlnfsX2	c.1218_1231del14ins12	D	no	no
4	*PCCB*	p.Glu331X	p.Glu331X	C	no	yes
5	*PCCB*	p.Glu331X	p.Glu331X	D	yes	yes
6	*PCCB*	p.Arg514X	p.Arg529ArgfsX44		yes	
7	*PCCB*	p.Arg165Trp	?	B	no	
8	*PCCB*	p.Phe180Ser	p.Phe180Ser		yes	yes
9	*PCCB*	p.His258Arg	p.Arg376Cys	B	no	no
10a	*MMAA*	del exon2-3-4	del exon2-3-4	A	no	
10b	*MMAA*	del exon2-3-4	del exon2-3-4	D	yes	yes
11	*MMAA*	p.Arg22X	p.Arg22X	A (6 y)	no	yes
12	*MMAA*	p.Thr221del	p.Thr221del	A	no	
13	*MMAA*	p.Arg145X	?	A	no	
14	*MMAA*	p.Arg196X	p.Arg196X	A (6 y)	no	
15	*MMAA*	c. 592_595del	c.594_598del	A	no	no
16	*MMAA*	p.Lys276Asn	p.Lys276Asn	A	no	
17	*MMAB*	p.Arg186Trp	p.Arg190Cys	A (6 y)	no	
18	*MUT*	c.360_361insT	c.360_361insT	C	no	yes
19	*MUT*	c.693_694insA	c.693_694insA	D (6 y)	no	yes
20	*MUT*	p.Gln352X	p.Gln352X		no	
21	*MUT*	p.Arg474X	p.Arg511X	C	yes	yes
22	*MUT*	c.1876_1888del	c.1876_1888del	D	yes	yes
23	*MUT*	p.Arg228X	p.Arg369Cys		yes	no
24	*MUT*	p.Ser342X	p.Arg93His	B (6 y)	no	
25	*MUT*	p.Ser342X	p.Arg694Trp	C	no	no
26	*MUT*	p.Ser342X	p.Ala191Glu	A (6 y)	no	yes
27	*MUT*	c.662delT	p.Gly158Val		no	no
28	*MUT*	p.Arg511X	p.Gly642Arg	A	no	no
29	*MUT*	p.Arg511X	p.Arg603Thr	D (6 y)	no	no
30	*MUT*	p.Ala191Glu	p.Ala191Glu	D (6 y)	no	yes
31	*MUT*	p.Ala191Glu	p.Ala191Glu	D	no	
32	*MUT*	p.Ala191Glu	p.Ala191Glu	A (6 y)	no	yes
33a	*MUT*	p.Ala191Glu	p.Gly623Arg	A (6 y)	no	
33b	*MUT*	p.Ala191Glu	p.Gly623Arg	A	yes	no
34	*MUT*	p.Gly203Arg	p.Arg108Cys		no	yes
35	*MUT*	p.Gly203Arg	p.Met700Lys	A	no	no
36	*MUT*	p.Gly215Ser	p.Gly215Ser	A (6 y)	no	no
37	*MUT*	p.Asn219Tyr	p.Asn219Tyr	A	no	no
38	*MUT*	p.Asn219Tyr	p.Asn219Tyr		yes	no
39	*MUT*	p.Asn219Tyr	p.Gln383His	D	yes	yes
40	*MUT*	p.Asn219Tyr	p.Val162Phe	B (6 y)	no	
41	*MUT*	p.Asn219Tyr	p.Ala191Glu	D	yes	yes
42	*MUT*	p.Thr230Ile	p.Thr230Ile	A	no	yes
43a	*IVD*	p.Arg284Gln	p.Arg284Gln		no	
43b	*IVD*	p.Arg284Gln	p.Arg284Gln	A	no	
44	*IVD*	p.Gly289Arg	p.Gly289Arg		no	yes
45	*IVD*	p.Cys378Arg	p.Cys378Arg	A	no	
46	*IVD*	p.Arg392His	p.Arg392His	B	no	

For PA patients, there was no correlation between genotype and neurological evolution, especially between patients with two stop mutations and those with two missense mutations. The number of patients was not sufficient for any in-depth investigation.

For MMA patients, cognitive prognosis and neurological evolution were significantly better at age 6 or 11 years, for *MMAA* mutated patients compared to patients with mutation in *MUT*. Indeed, 7/8 *MMAA* mutated patients and 9/26 *MUT* mutated patients were classified in the group A (p < 0.05, Fisher's exact test) while 1/8 *MMAA* mutated patients and 7/26 MUT mutated patients had abnormal neurological examination. Furthermore, cognitive evolution at 6 or 11 years old seemed to be better for patients with mutation in *MUT* with two missense mutations (7/12 in group A) compared to those with two stop mutations (four patients in group C or D). However there was quite heterogenous neurological prognosis for patients with the same mutation.

We found no genotype phenotype correlation in IVA patients.

## Discussion

This study investigates the long-term neurological prognosis of 80 patients presenting with classical organic acidurias, i.e. PA, MMA and IVA that involve the branched-chain amino acids metabolism. The results reinforce the notion that PA patients have the most severe neurological prognosis with 37% of patients with abnormal neurological examination, 61% with abnormal cognitive test at age three years and 56% with basal ganglia lesions. PA patients also presented more frequent acute decompensations from zero to three years old (1.8 on average). Four patients had psychiatric troubles including delirious syndrome during an acute decompensation. However, long-term cognitive evaluation was similar to MMA because of an improvement of psychometric tests with 47% of PA patients achieving normal evaluation at 11, which was not paralleled by MMA patients. Recently, Grünert et al. [5] reported a cohort of 55 PA patients with intellectual deficiency in 75% of patients (median IQ at 55), hypotonia in 51% of patients and ataxia in 9% of patients. Brain MRI revealed basal ganglia lesions in 5/26 patients [5]. We note that our data correspond to a psychometric evaluation performed at age 11 years. Most patients reported by Grünert et al. were younger (median age: 5.2 years [5]), which may explain why they did not (yet!) show the cognitive improvement that we have observed on average. Nevertheless in some of our patients, evolution rather involved a progressive neurological worsening.

IVA had the best neurological prognosis with normal neurological examination in all patients, though white matter anomalies were noted in 3 patients of 6 available MRI. However, fine motor trouble was detected in 22% of patients, as well as some degree of intellectual deficiency in one third of IVA patients. Our data complement a study realized on 16 IVA patients including 44% with coordination trouble or moderate motor trouble and 16% with intellectual deficiency [2]. In literature, neurological prognosis was good for 85% of IVA patients diagnosed during neonatal period while only for 45% of the patients diagnosed later. Eventually, some acute decompensations occurred during teenage years.

MMA had an intermediate prognosis between PA and IVA with 24% of abnormal neurological examination, 54% of brain anomalies and 50% of intellectual deficiency at 11 years old.

Identification of prognostic factors would be useful to prevent neurological complications and to guide long-term follow up. Early diagnosed patients had better neurological prognosis as neurological examination was normal for 87% of patients diagnosed during neonatal period but only 66% of those diagnosed later (p < 0.05), as previously described in several studies, arguing in favor of neonatal screening for organic acidurias [7-9]. Most forms being revealed at a few post-natal days of life, neonatal screening will be effective for later onset forms. In this cohort, all patients with an extra-pyramidal syndrome were diagnosed after the neonatal period. By contrast, the number of acute decompensations and their severity did not influence the prognosis. Similarly, there is no correlation between brain anomalies and abnormal neurological examination or intellectual deficiency. Likewise, liver transplants for PA and MMA and kidney or combined liver-kidney transplant for MMA improve the metabolic status. Benefits are shown in term of significant improvement of quality of life with increased protein tolerance, reduction of acute decompensations and hospitalizations. However transplantation does not cure the disease. Metabolic decompensations are less common but remain possible and long-term complications, in particular neurological impairment, are not prevented [10]. This suggests that additional prognostic factors are involved, notably the degree of metabolite balance in the basal state. Considering standard metabolites, there was no correlation between the biochemical profile excluding the acute decompensations and neurological outcome. However, we found significant progressive increases of the three plasma amino acids tested in PA and not in IVA, with an intermediate pattern in MMA (Table 1 and Figure 8). Because the deviating plasma amino acid pattern progresses with age, it might result from unsatisfactory management during follow-up rather than at diagnosis. This may be relevant to the finding that intellectual ability in our PA cohort is better at 11 years than reported at an earlier age in another cohort (see above) [5]. Thus, greater efforts may be needed to optimize long-term management in these patients.

Considering patients with MMA, plasma methylmalonic acid levels were higher in patients with intellectual deficiency, with an “alert” value estimated at 167 μmol/L. In the same way, urinary 3-hydroxypropionate level was higher in patients with intellectual deficiency, with an “alert” value of 55 μmol/mmol of creatinine. Finally, genotype analysis was not informative for prognosis. As for PA, there was no genotype phenotype correlation presumably because of the small number of patients. Previous studies reported that the phenotype was more severe among patients with two stop mutations or with missense mutations altering protein stability, especially those involving *PCCB*[11,12]. As for MMA, global neurological prognosis was better for patients with mutations in *MMAA* gene compared to those mutated in *MUT* gene even if most mutations in *MMAA* were stop mutations. These results can be explained by the role of the protein coded by *MMAA* in synthesis of adenosylcobalamin, the cofactor of methylmalonyl-CoA mutase, and its occasional sensitivity to B12 supplementation. Consistent data have already been presented in previous studies [13,14]. As for IVA, molecular data were available for only five patients and there was no obvious genotype phenotype correlation. A previous study on a cohort of patients presenting with the same homozygous missense mutations showed great phenotypic variability [6].

Finally, clinical and biochemical differences observed between the three organic acidurias highlighted some differences of pathophysiological relevance. The best prognosis was associated with IVA, possibly because of a great efficacy of glycine to detoxicate accumulated metabolites. Furthermore, leucine degradation leads to acetyl-CoA and not succinyl-CoA formation contrary to PA and MMA, making the energy defect possibly less pronounced in IVA. This is also suggested by the absence of basal ganglia lesions and the normal lactate peak on magnetic resonance spectroscopy. An *in vitro* study on rat cortex synapses showed that IVA metabolites do not modify tricarboxylic cycle and respiratory chain activity but inhibits Na^+^,K^+^-ATPase, an enzyme playing a role in brain development and neuronal stability [15]. Phenotypic variability observed in a homogenous population suggests that other environmental or genetic factors modify isovaleryl-CoA deshydrogenase activity or the efficiency of glycino-conjugation [6]. Other metabolites, such as isovalerylglutamate, methylfumarate, isovalerylserine and 3-hydroxycaproate have recently been detected and could play a role in pathophysiology [16]. By contrast, PA generates several toxic metabolites such as 3-hydroxypropionate, methylcitrate, and propionyl-CoA. We have shown that urinary 3-hydroxypropionate level correlates with long term neurologic outcome consistent with previous hypotheses based on propionate toxicity on glial cell metabolism [17]. Propionyl-CoA and methylcitrate are responsible for mitochondrial dysfunction by enzyme inhibition and propionyl-CoA decreases acetyl-coA formation by CoA sequestration [18,19]. These features may account for the severity of PA deficiency that frequently leads to basal ganglia lesions, high lactate peak on magnetic resonance spectroscopy and significant increase of plasma alanine level. Furthermore, increased plasma glycine is more pronounced in PA patients thus also arguing for an important dysfunction of the glycine cleavage system in mitochondria. In MMA, methylmalonic acid levels also correlated with long-term neurological outcome. Other metabolites such as 3-hydroxypropionate, methylcitrate, tiglyglycine and propionylgycine were present in much smaller amounts compared to PA. The intermediate prognosis of MMA suggests that toxicity may mainly result from intermediate levels of PA metabolites. Indeed, it has been demonstrated that methylmalonate has no obvious toxicity on respiratory chain [20], yet secondary respiratory chain deficiency has been reported in PA and MMA patients in different tissues [21,22].

## Conclusion

In conclusion, this study of 80 patients with organic acidurias brings additional knowledge on long-term neurological outcome and highlights the need to identify relevant environmental or genetic prognostic factors that may modulate severity of the disorders. We have identified prognostic “alert” values for the two more severe deficiencies, MMA and PA. Our clinical, radiological and biochemical data strengthen the hypothesis of a mechanism of direct mitochondrial toxicity for PA and to a smaller degree, for MMA.

## Competing interests

The authors declare that they have no competing interests.

## Authors’ contributions

MN designed the study, performed it and wrote the manuscript; CO performed statistical and biochemical analysis and participated in the manuscript preparation; VV, JBA and GT described patient phenotype; VB performed psychometric tests; FH and DR performed biochemical analysis; ID and NB described neurological phenotype; JPB, CA and JFB performed molecular analysis; PdL designed the study and participated in the manuscript preparation. All authors read and approved the final manuscript.

## Supplementary Material

Additional file 1 **Average plasma amino acid levels +/- standard deviation according to age by each disease.** All available tests were included (at least one per year) except those obtained during acute decompensations. PA: propionic aciduria, MMA: methylmalonic aciduria, IVA, isovaleric aciduria. Click here for file
