# Supplementary material for: Long-term neurological outcome of a cohort of 80 patients with classical organic acidurias
Source: Orphanet J Rare Dis. 2013 Sep 23;8:148. doi: 10.1186/1750-1172-8-148 (PMC4016503; doi:10.1186/1750-1172-8-148)
Supplement: Additional file 1 — Average plasma amino acid levels +/- standard deviation according to age by each disease. All available tests were included (at least one per year) except those obtained during acute decompensations. PA: propionic aciduria, MMA: methylmalonic aciduria, IVA, isovaleric aciduria. [file 1750-1172-8-148-S1.pdf]

Additional file 1: Average plasma amino acid levels +/- standard deviation according to age by each disease. All available tests were included (at least one per year) except those obtained during acute decompensations. PA: propionic aciduria, MMA: methylmalonic aciduria, IVA, isovaleric aciduria.

	**Plasma glutamine (µmol/L)**			**Plasma glycine (µmol/L)**			**Plasma alanine (µmol/L)**		
	**PA**	**MMA**	**IVA**	**PA**	**MMA**	**IVA**	**PA**	**MMA**	**IVA**
**0-3 y**	515 +/- 96	562 +/- 98	448 +/- 82	831 +/- 297	434 +/- 163	551 +/- 104	434 +/- 107	450 +/- 120	481 +/- 112
**3-6 y**	638 +/- 109	583 +/- 109	375 +/- 91	1143 +/- 354	643 +/- 324	489 +/- 100	471 +/- 116	506 +/- 148	377 +/- 63
**6-11 y**	621 +/- 93	604 +/- 110	425 +/- 129	1485 +/- 539	599 +/- 184	561 +/- 114	523 +/- 110	518 +/- 98	410 +/- 67
**> 11y**	760 +/- 166	639 +/- 108	463 +/- 77	1472 +/- 389	681 +/- 293	568 +/- 71	652 +/- 164	597 +/- 147	435 +/- 102
